# First person – Lauren Brilli Skvarca and Hwa Han

**DOI:** 10.1242/dmm.039859

**Published:** 2019-04-05

## Abstract

First Person is a series of interviews with the first authors of a selection of papers published in Disease Models & Mechanisms (DMM), helping early-career researchers promote themselves alongside their papers. Lauren Brilli Skvarca and Hwa Han are co-first authors on ‘Enhancing regeneration after acute kidney injury by promoting cellular dedifferentiation in zebrafish’, published in DMM. Lauren is a Pathologist Investigator Residency Research Training (PIRRT) Fellow at the University Pittsburgh Medical Center (UPMC), USA, in the lab of Carl Hubel, investigating maternal-fetal cell interactions contributing to placental vascular changes in preeclampsia and postpartum maternal cardiovascular risk. Hwa is a PhD graduate student in the lab of Neil Hukriede at the University of Pittsburgh School of Medicine, USA, and is involved in characterizing regenerative cellular mechanisms in gentamicin-induced acute kidney injury using larval zebrafish as a model organism.


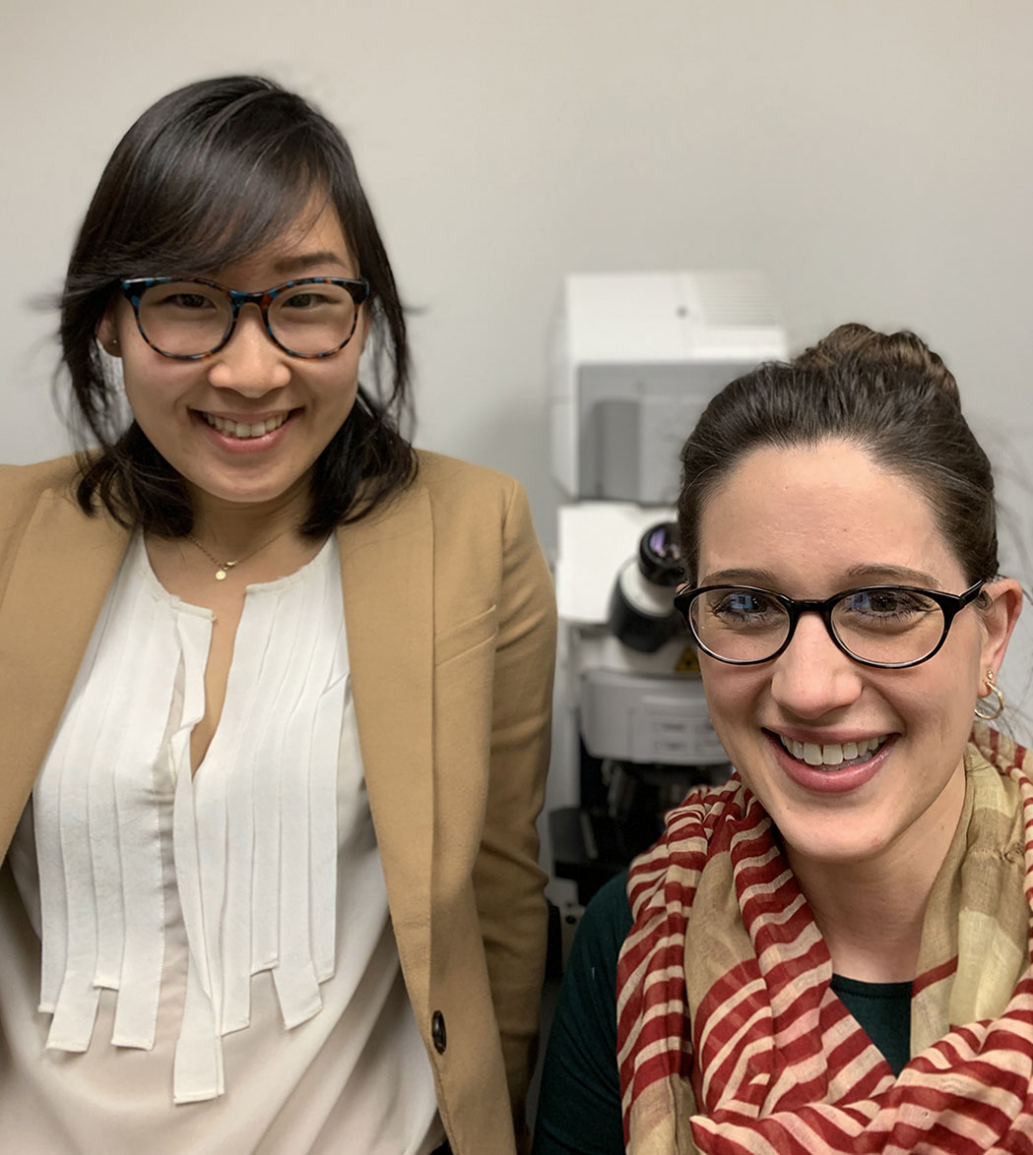


**Hwa Han and Lauren Brilli Skvarca**

**How would you explain the main findings of your paper to non-scientific family and friends?**

LBS+HH: The goal of our work is to understand how the kidney repairs itself after injury so that we can harness these innate mechanisms to develop better treatment options for people who experience acute kidney injury. This disease occurs more often in hospitalized patients than outpatients and has many causes, ranging from dehydration to infection. Drugs are another frequent cause of injury, although many of these drugs are necessary to treat serious conditions such as cancer or transplant rejection. These sometimes non-preventable causes of injury emphasize the need to find novel ways to enhance kidney regeneration. In our study, we used zebrafish to model acute kidney injury because its developing renal system harbors key similarities to the adult human kidney at the cellular level. We utilized zebrafish strains that allowed us to track multiple factors in the injury-repair pathway after delivery of PTBA, a compound that shows promise as a potential treatment for acute kidney injury. In kidney cells, we found that UPHD25 (PTBA prodrug) treatment reduced markers of injury and increased markers of repair. PTBA also altered the quantity and type of immune repair cells present in the kidney after injury. Our data suggest that these effects are mediated by retinoic acid signaling, a pathway known for its involvement in both organ development and regeneration in multiple contexts. This study provides insight into how PTBA works at the cellular level by identifying signaling pathways important for kidney repair.

**What are the potential implications of these results for your field of research?**

LBS+HH: We demonstrated that PTBA increases proliferation and dedifferentiation of proximal tubule cells in the zebrafish kidney. This complements previous work from our group and collaborators, which showed that PTBA increases renal function and ameliorates fibrosis in other kidney injury models, by delving deeper into the cellular mechanisms. Together, these results suggest that PTBA is a compelling candidate for further study. This is particularly important because no drugs are currently available to patients who experience acute kidney injury, so our work takes an exciting step toward expanding treatment options. However, much work is still required. Our current hypothesis is that PTBA acts as a histone deacetylase inhibitor, so our focus now is to identify target proteins and downstream pathways that may be critical in the pathophysiology of acute kidney injury.

“Our work takes an exciting step toward expanding treatment options. However, much work is still required.”

**What are the main advantages and drawbacks of the model system you have used as it relates to the disease you are investigating?**

LBS: The pronephric larval zebrafish kidney is composed of only two nephrons. The simplicity of this system likely impairs the organism's ability to recover globally after kidney injury. So acute kidney injury is often fatal in our model, in contrast to mammals, which generally recover. However, this simplicity is also a strength. The entire renal system is contained within a small, optically clear organism that makes it ideally suited for time-lapse live imaging studies. For example, in our study, we performed live imaging in transgenic strains to track immune cells labeled with fluorescent proteins after kidney injury. This allowed us to characterize the immune cell milieu in proximity to each nephron and examine how the environment changed during the injury-repair sequence.

**Figure:**
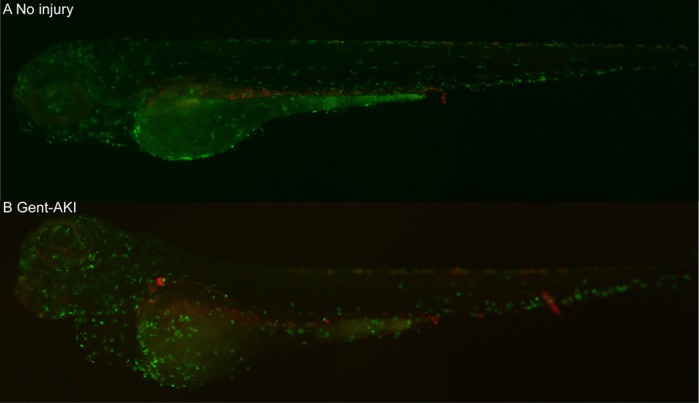
**Fluorescent image of *Tg(mpeg1:dendra2/cdh17:mcherry)*, a double-transgenic line used to capture real-time macrophage response in zebrafish during acute kidney injury.** The transgenic line expresses reporter fluorophores for macrophages (green) and pronephric kidney (red). No injury group shows steady-state macrophage circulation in the larva (A), while the injured group (Gent-AKI; B) shows increased Dendra2+ macrophages particularly concentrated in the pericardial and proximal tubules.

HH: Using zebrafish also affords advantages in the context of drug discovery. Due to their small size and rapid development, embryos and larvae are well suited for high-throughput studies to test large compound libraries for effects on development and regenerative capacity in the whole organism. Our lab previously performed these screening experiments, and this ultimately led to the identification of PTBA as a compound that can enhance renal recovery after injury. The simplicity and the small size of larvae also results in disadvantages. For example, established blood or urine assays that estimate renal function and recovery in mammals (e.g. blood urea nitrogen and serum creatinine) are not currently possible in our model system. However, this disadvantage has created opportunities to collaborate with groups that use mouse renal injury models, which will facilitate a more comprehensive understanding of how PTBA affects long-term renal recovery.

**What has surprised you the most while conducting your research?**

HH: I was surprised by our real-time two-photon videos capturing macrophage interaction with proximal tubules. It showed stark differences in macrophage response between injured and uninjured groups – macrophages migrated to the site of injury and had prolonged interaction with the tubules while undergoing phagocytosis of damaged renal cells. To my knowledge, this is the first effort to capture the immune response during acute kidney injury in real time, and I was excited to use zebrafish as a model system to demonstrate this finding.

LBS: I was most surprised and excited by our results demonstrating that PTBA may affect common regenerative pathways in two different injury models: acute kidney injury in zebrafish larvae and cardiac amputation injury in adult zebrafish. Retinoic acid signaling is critical during both kidney and heart development, and our findings suggest that PTBA may synergize with this pathway during repair. Together, these data broaden the applicability of PTBA as a potential treatment strategy to promote cellular regeneration post-injury in other organs.

**Describe what you think is the most significant challenge impacting your research at this time and how will this be addressed over the next 10 years?**

LBS: In order to move this work forward, we need to further identify specific PTBA protein and gene targets. Since PTBA likely inhibits histone deacetylases, it will be important to characterize its effects at the levels of transcription and epigenetics to understand its function and predict off-target effects. These efforts are currently underway in Dr Hukriede's lab.

On a global scale, I think the scientific and medical communities currently face growing public skepticism, which shows in ways ranging from hesitation to participate in clinical trials, to anti-vaccination campaigns, and ultimately to lack of support for research funding. I think this provides a great opportunity to reflect on our collective responsibility to share science in more accessible ways. Now is the perfect time for us to come together and rebrand as ambassadors dedicated to improving global health, one scientific discovery at a time. Rising to this challenge will require effective communication strategies to reach people of all ages and backgrounds, whether through elementary school outreach programs or meet-and-greet-your-scientist events in our communities. I view my scientific career as an incredible way to play a small part in healthcare innovation, and opportunities like this to share our work and passion on a more personal level are a great way to make science more approachable to a broader audience.

HH: Zebrafish in the realm of pathophysiology research is often under attack because zebrafish are deemed ‘unfit’ due to perceived lack of conservation. Every model organism has its advantages and disadvantages. Scientists in each domain can conduct research keeping in mind how best to use their model organism to strengthen their findings. For example, zebrafish is an excellent and unique tool in tracking real-time cell-to-cell interaction in a system with all critical organ systems intact. In the kidney field, a growing number of groups are developing human kidney organoid system for various utilities, such as drug screening and disease modeling. Zebrafish research will remain important in its own right, but the findings could be complemented with human organoids by validating targets in the human genome and proteome.

**What changes do you think could improve the professional lives of early-career scientists?**

HH: As Lauren mentioned, we live in a political climate where scientific funding is ever-so-declining, academic positions are competitive, and the public's trust in science is in decline. For a scientist to be successful in such an environment, PhD recipients should walk out of their graduate programs with more than just a degree. It calls for a well-rounded training. The training needs to equip scientists with the ability to apply our scientific problem-solving skills to real-world situations that can be put on résumés. This means graduate programs widening their offerings to allow for externships, whether it be in business, scientific writing, medicine, public communication or teaching.

LBS: Building on Hwa’s point, I think young scientists should be encouraged to build collaborations across fields – and even outside the field of science. While it is of course important to network within one's subspecialty, staying exclusively within that comfort zone can be limiting. Mentors with broad expertise challenge you to think about research from different perspectives. You never know what direction your next set of unexpected results may take you, so it is empowering to have a wide range of expertise to fall back on! My hope is that this approach would foster a sense of connection to the entire scientific community and encourage enthusiasm among trainees to continue their careers in the field.

“I think young scientists should be encouraged to build collaborations across fields – and even outside the field of science.” *– Lauren Brilli Skvarca*

**What's next for you?**

LBS: After finishing my PhD in Dr Hukriede's lab and completing medical school, I began graduate medical residency training in anatomic pathology at UPMC in Pittsburgh, USA. One theme of my thesis work that stuck with me is the importance of immune cell interactions with other cell types to coordinate a response. This applies to multiple contexts ranging from kidney tubule epithelial cell repair to cancer cell metastasis. This common theme has led me to the ‘ultimate’ cell interface between mother and fetus in the developing placenta. My current scientific project in Dr Carl Hubel's lab at Magee-Women's Research Institute examines how problems at this placental interface lead to adverse pregnancy outcomes. Specifically, my project focuses on understanding how impaired maternal vascular adaptation during placental development leads to preeclampsia and increases maternal risk of developing cardiovascular disease postpartum. My career goal is to pursue these interests as a future academic perinatal pathologist.

HH: I plan on obtaining my PhD in Molecular Genetics and Developmental Biology at University of Pittsburgh School of Medicine. I am incredibly fortunate to be in a laboratory where I can explore post-PhD career options while doing benchwork. I am also fortunate to be in an institution where I can observe research that direct impacts patient care. Along with my laboratory benchwork, I've had a great opportunity to volunteer in a clinic and have patient contact. I will be applying to medical schools upon completion of my PhD to start new training as a physician.
